# CDP7657, an anti-CD40L antibody lacking an Fc domain, inhibits CD40L-dependent immune responses without thrombotic complications: an *in vivo* study

**DOI:** 10.1186/s13075-015-0757-4

**Published:** 2015-09-03

**Authors:** Anthony Shock, Linda Burkly, Ian Wakefield, Christopher Peters, Ellen Garber, Janine Ferrant, Frederick R. Taylor, Lihe Su, Yen-Ming Hsu, David Hutto, Ali Amirkhosravi, Todd Meyer, John Francis, Sarah Malcolm, Martyn Robinson, Derek Brown, Stevan Shaw, Roland Foulkes, Alastair Lawson, Olivier Harari, Timothy Bourne, Alison Maloney, Neil Weir

**Affiliations:** UCB Pharma, 216 Bath Road, Slough, Berkshire SL1 4EN UK; Biogen Idec, Inc., 12 Cambridge Center, Cambridge, MA 02142 USA; UCB Pharma, Braine, Belgium; Center for Thrombosis Research, Florida Hospital, Orlando, USA; Present Address: Ab Biosciences Inc., Allston, MA USA; Present Address: Charles River, Wilmington, MA USA; Present Address: Cheylard Biosciences, Berkshire, UK; Present Address: Hammel Ltd, Buckinghamshire, UK

## Abstract

**Introduction:**

CD40 ligand (CD40L) blockade has demonstrated efficacy in experimental autoimmune models. However, clinical trials of hu5c8, an anti-human CD40L IgG_1_ antibody, in systemic lupus erythematosus (SLE) were halted due to an increased incidence of thrombotic events. This study evaluated CDP7657, a high affinity PEGylated monovalent Fab' anti-CD40L antibody fragment, to assess whether an Fc-deficient molecule retains efficacy while avoiding the increased risk of thrombotic events observed with hu5c8.

**Methods:**

The potency and cross-reactivity of CDP7657 was assessed in *in vitro* assays employing human and non-human primate leukocytes, and the capacity of different antibody formats to activate platelets *in vitro* was assessed using aggregometry and dense granule release assays. Given the important role CD40L plays in regulating humoral immunity, *in vivo* efficacy was assessed by investigating the capacity of Cynomolgus monkeys to generate immune responses to the tetanus toxoid antigen while the potential to induce thrombotic events *in vivo* was evaluated after repeat dosing of antibodies to Rhesus monkeys. A PEGylated anti-mouse CD40L was generated to assess efficacy in the New Zealand Black/White (NZB/W) mouse model of SLE.

**Results:**

CDP7657 dose-dependently inhibited antigen-specific immune responses to tetanus toxoid in Cynomolgus monkeys, and in contrast to hu5c8, there was no evidence of pulmonary thrombovasculopathy in Rhesus monkeys. Aglycosyl hu5c8, which lacks Fc receptor binding function, also failed to induce thrombotic events in Rhesus monkeys. *In vitro* experiments confirmed that antibody constructs lacking an Fc, including CDP7657, did not induce human or monkey platelet activation. A PEGylated monovalent Fab' anti-mouse CD40L antibody also inhibited disease activity in the NZB/W mouse model of SLE after administration using a therapeutic dosing regimen where mice received antibodies only after they had displayed severe proteinuria.

**Conclusions:**

These findings demonstrate for the first time that anti-CD40L antibodies lacking a functional Fc region do not induce thrombotic events in Rhesus monkeys and fail to activate platelets *in vitro* but, nevertheless retain pharmacological activity and support the investigation of CDP7657 as a potential therapy for systemic lupus erythematosus and other autoimmune diseases.

**Electronic supplementary material:**

The online version of this article (doi:10.1186/s13075-015-0757-4) contains supplementary material, which is available to authorized users.

## Introduction

CD40 ligand (CD40L), or CD154, is expressed on activated T lymphocytes, and through interactions with its receptor CD40, plays a pivotal role in regulating the interplay between T cells and other cell types [1–3]. The CD40L/CD40 pair is known to mediate cognate T cell help for B cells, resulting in increased B cell proliferation and differentiation, antibody production and isotype class switching. CD40L also promotes the formation of germinal centers in lymph nodes and B-cell survival. CD40L therefore holds significant promise as a therapeutic target in autoimmune disease, and blockade of CD40L has been shown to be highly efficacious in several inflammatory and autoimmune model systems [4–8].

CD40L has also been detected on other immune and non-lymphoid cells [3] and is present in platelets [9, 10]. Within seconds of being stimulated by platelet agonists, CD40L is presented on the surface of platelets, and is subsequently shed as soluble CD40L (sCD40L) [9]. Furthermore, a role for platelet-derived CD40L in regulating adaptive immunity and thrombosis has been suggested [11–14].

Hu5c8, a monoclonal IgG_1_ antibody against CD40L, was evaluated in clinical trials for a range of autoimmune diseases. Results from a phase 2 study in patients with systemic lupus erythematosus (SLE) were encouraging, with significant reductions in disease biomarkers, including circulating levels of autoantibodies and marked increases in C3 levels [15–17]. However, despite this promising evidence of clinical effect, further development of hu5c8 was discontinued because of an increased incidence of treatment-emergent cardiovascular thrombotic events (TEs) [18]. More recently, in a study of hu5c8 in the Rhesus monkey, numerous TEs including pulmonary vascular thrombi and vasculopathy were found after administration of hu5c8 [19], suggesting that the Rhesus monkey is a relevant and sensitive pre-clinical model for induction of TEs by anti-CD40L IgG_1_ antibodies in humans. The mechanism by which hu5c8 induces TEs in humans remains unclear. *In vitro* analyses have shown that immune complexes (IC) consisting of sCD40L and an anti-CD40L monoclonal antibody can trigger platelet aggregation [12, 20]. This effect seems to be dependent on the anti-CD40L monoclonal antibody carrying a functional Fc region, and signaling through the FcγRIIa (CD32a) Fc receptor on the platelet surface [12, 20, 21].

To evaluate whether one could achieve the therapeutic potential of CD40L blockade while removing the TE risk hypothesized to be due to platelet activation, this paper describes the generation and testing of an anti-CD40L antibody fragment lacking a functional Fc region. To increase the circulating half-life of this monovalent Fab' antibody fragment, a 40 kDa polyethylene glycol (PEG) moiety was conjugated via a chemical linker, and this new molecule was designated CDP7657. CDP7657 is currently undergoing phase 1 clinical trials in patients with SLE.

A series of experimental studies was conducted utilizing a variety of antibody constructs, including CDP7657 and aglycosyl IgG forms of anti-CD40L antibodies, in order to determine the importance of Fc function and valency for the efficacy and safety of anti-CD40L antibodies. Here, we describe findings demonstrating that CDP7657 inhibits antigen-specific immune responses to the tetanus toxoid (TT) antigen in Cynomolgus monkeys. The profound efficacy of an anti-mouse CD40L monovalent PEGylated Fab' fragment was also demonstrated in a murine model of SLE. In addition, the Fc region of anti-CD40L antibodies was shown to be important for the activation of platelets *in vitro* and for the induction of TEs in Rhesus monkeys *in vivo*.

## Methods

### Antibodies and reagents

Anti-CD40L antibodies used were: hu5c8, a humanized anti-human CD40L intact IgG_1_ monoclonal antibody (Biogen Idec, Cambridge, MA, USA); aglycosyl hu5c8, a mutant form of hu5c8 with reduced FcR binding (Biogen Idec, MA, USA); CDP7657, a monovalent Fab' PEGylated anti-CD40L antibody (UCB Pharma, Slough, UK); M90, a mouse anti-human CD40L intact IgG_1_ monoclonal antibody (hybridoma from ATCC, Manassas, VA, USA); MR1 Fab' PEG, a monovalent, murinized Fab' PEGylated anti-mouse CD40L (UCB Pharma, Slough, UK). Soluble CD40L was from Peprotech (Rocky Hill, NJ, USA), phycoerythrin (PE)-labeled CD40-Fc fusion protein was from Ancell (Bayport, MN, USA) and thrombin receptor agonist peptide (TRAP) was from Sigma (St Louis, MO, USA).

### Activity and cross-reactivity experiments

BIAcore (surface plasmon resonance) experiments were performed to calculate the affinity (K_D,_ equilibrium dissociation constant) of CDP7657 for CD40L and involved capturing the antibody using an anti-human Fab' antibody and then titrating human sCD40L protein over the immobilized antibodies. The K_D_ for hu5c8 was assessed after capture with an anti-Fc antibody. Potency was assessed in two flow cytometry-based, cell-based assays: the binding of a PE-labeled CD40–Fc fusion protein to CD40L-expressing D1.1 Jurkat cells and an assay of T-cell-dependent B cell activation, involving overnight co-culture of CD40L-expressing D1.1 cells with the Ramos B cell line and monitoring intercellular adhesion molecule 1 (ICAM-1) expression on the latter cells. Non-human primate cross-reactivity of CDP7657 was measured in saturation binding experiments using a broad-range antibody titration on peripheral blood lymphocytes isolated from humans, Rhesus monkeys and Cynomolgus monkeys and activated *in vitro* to express CD40L. Human blood samples were obtained under a licence (#12504) granted to UCB under Section 16 (2) (e) (ii) of the Human Tissue Act (UK) and all donors provided their written consent to participate.

### Immune response to TT in a Cynomolgus monkey model

Groups of six Cynomolgus monkeys (n = 3/sex) were administered a single intravenous (i.v.) dose of antibody or saline and, after 4 h or 48 h, an intramuscular (i.m.) dose of TT (0.5 mL), except in the dose–response study, where 20 mg/kg IgG antibodies and 40 mg/kg PEGylated antibodies were used. Plasma was isolated at various time intervals for anti-TT analyses by ELISA. Plates were coated with TT, incubated with plasma samples and after washing, binding was detected using horseradish peroxidase (HRP)-conjugated anti-monkey IgG antibodies. Mean anti-TT IgG titer ± standard deviation were calculated and where appropriate, one-way analysis of variance (ANOVA) was performed. As parametric statistical methods were used, the data should be declared to have been found normally distributed and of equal variance.

### NZB/W SLE nephritis mouse model

All experiments were conducted under protocols approved by the Animal Welfare and Ethical Review Body of UCB (UK) in accordance with the Animals (Scientific Procedures) Act 1986. Female NZB/NZW F_1_ mice (Harlan, UK) aged 18 weeks, were housed in cages of seven or eight mice, in a temperature- and humidity-controlled room, with 12-h light–dark cycles. All animals received food and water *ad libitum*.

Female NZB/NZW F_1_ mice were assessed for disease progression from 18 weeks of age with levels of protein (mg/dL) in the urine measured by Albustix (Bayer, Leverkusen, Germany) twice a week. After recording a proteinuria level ≥300 mg/dL on one occasion, mice were randomized into one of two dosing groups and dosed subcutaneously (s.c.) with either saline or 100 mg/kg MR1 Fab' PEG. Animals entered disease remission when a proteinuria level ≤100 mg/dL was measured on two consecutive occasions, and disease relapse was classified as animals that scored ≥300 mg/dL on two consecutive occasions. Remission and relapse data were subjected to the Mann–Whitney *U* test to compare MR1 Fab' PEG to saline control. Survival data were analyzed by the log-rank test.

### Platelet activation by anti-CD40L antibodies

#### Platelet aggregometry

The protocol for obtaining human blood for all platelet studies was approved by the Florida Hospital Institutional Review Board and donors provided their written consent to participate. Washed platelet aggregation studies were performed as described previously [22]. Briefly, washed platelets (four human donors, two Rhesus monkeys) were suspended in HEPES buffer, and platelets were adjusted to 250/nL and analyzed by light transmission aggregometry (300 μL/assay). Baseline traces were established and preformed anti-CD40L ICs were added to a final concentration of 200 nM. For studies with preformed IC, antibody formats (hu5c8, aglycosyl hu5c8 or CDP7657) were combined with sCD40L in a ratio of one antibody to one antigen homotrimer, the molar stoichiometric equivalent of this being one antibody per three sCD40L antigen monomers. Aggregations were monitored for at least 6 minutes.

#### Measurement of platelet dense granule release

The serotonin release assay was performed as described previously [21]. Briefly, platelets in platelet-rich plasma (one human donor, four Rhesus monkeys) were labeled with ^14^C-radiolabeled serotonin (0.1 μCi/mL), incubated for 45 minutes at 37 °C, washed in apyrase buffer (to prevent spurious activation), then re-suspended in albumin-free Tyrode’s assay buffer. Antibody solutions were clarified by centrifugation (16,100 rpm, 15 minutes) before adding to assay wells to a final concentration of 500 nM. For studies with preformed IC, antibody formats (hu5c8, aglycosyl hu5c8 or CDP7657) were combined with sCD40L in a ratio of one antibody to one antigen homotrimer, the molar stoichiometric equivalent of this being one antibody per three sCD40L antigen monomers. ICs, pre-incubated for 5–20 minutes prior to assay, were then added to microtiter plate wells to achieve a final concentration of 500 nM.

### Assessment of TE in Rhesus monkeys

Studies in Cynomolgus and Rhesus monkeys were conducted at Charles River Laboratories (NV, USA), followed international Good Laboratory Practice (GLP) standards and were conducted in accordance with the regulations of the USDA Animal Welfare Act (2010) and in compliance with the testing facility’s Animal Welfare Assurance (A4112-01) filed with the National Institutes of Health (NIH); the protocols were approved by the Charles River Laboratories Institutional Animal Care & Use Committee.

A head-to-head comparative 8-week study investigated the occurrence of TEs with the monovalent Fab' PEG CDP7657 and two intact monoclonal antibodies, hu5c8 and aglycosyl hu5c8. These agents were administered i.v. at 50 mg/kg/week for 8 weeks to Rhesus monkeys (n = 4/sex/group) and compared with saline-injected controls (n = 2/sex).

A 3-month GLP pivotal safety study assessed potential dose-dependent TE occurrence with CDP7567 and general potential for toxicity. CDP7567 was administered at 20, 50 or 200 mg/kg/week i.v. (n = 5/sex/group) for 12 weeks. Control animals (n = 10/sex) received saline. At the end of the treatment period, additional satellite groups (n = 1 − 4/sex/group) were used to assess any reversibility effects over 4 and 6 months. For both studies, monoclonal antibodies were administered by bolus i.v. injection over approximately 60 s. Standard toxicology endpoints were monitored. At necropsy, lungs were insufflated with neutral-buffered formalin and allowed to fully fix by immersion in additional neutral-buffered formalin.

Macroscopic examination of pulmonary tissues included partial dissection of pulmonary and bronchial arteries. A total of 29 histopathology sections were taken from each animal. Five histopathology sections were collected from each of the four largest lung lobes (left and right diaphragmatic, and left and right apical). Three sections were prepared from the three smallest lung lobes (left and right middle lobes, and intermediate lobe).

## Results

### CDP7657 inhibits the humoral immune response

CDP7657 has a very high affinity for CD40L and potently neutralizes CD40L in functional cell-based assays, measuring CD40 binding to CD40L, and CD40L-dependent B cell activation (Table 1). It had similar affinity for human and non-human primate (Rhesus monkey and Cynomolgus monkey) CD40L (Table 2), which was expected as the CD40L protein sequences of Rhesus monkeys and Cynomolgus monkeys are identical, and share 98 % of their identity with human CD40L. CDP7657 possesses a higher affinity than hu5c8 and both antibodies have similar activities in cell-based assays (Table 1). CDP7657 does not bind to rodent CD40L and, therefore, it was necessary to investigate the *in vivo* efficacy of CDP7657 in non-human primates. The model employed monitored the impact of CDP7657 on the antibody response to TT in Cynomolgus monkeys, a model previously used to evaluate hu5c8 efficacy [5].

**Table 1:** Affinity and potency of CDP7657 and hu5c8

Assay	CDP7657	Hu5c8
BIAcore affinity (K_D_)	7.9 pM	33 pM
CD40 binding (IC_50_)	44 ng/mL (490 pM)	44 ng/mL (293 pM)
CD40L-dependent B-cell activation (ICAM-1 expression) (IC_50_)	83 ng/mL (920 pM)	38 ng/mL (253 pM)*

*Activity in this assay is very sensitive to the valency of anti-CD40L antibodies tested, and the IC_50_ for hu5c8 Fab is 977 ng/mL (n = 20). *CD40L* CD40 ligand, *ICAM* intercellular adhesion molecule 1

**Table 2:** CDP7657 cross-reactivity with human and non-human primate (Cynomolgus and Rhesus monkey) CD40L

Cell type	K_D_ (nM)					
	Human		Cynomolgus monkey		Rhesus monkey	
	Donor 1	Donor 2	Donor 1	Donor 2	Donor 1	Donor 2
PBMC	1.57	1.41	1.50	1.13	1.21	0.80

*PBMC* peripheral blood mononuclear cell

**Fig. 1:**
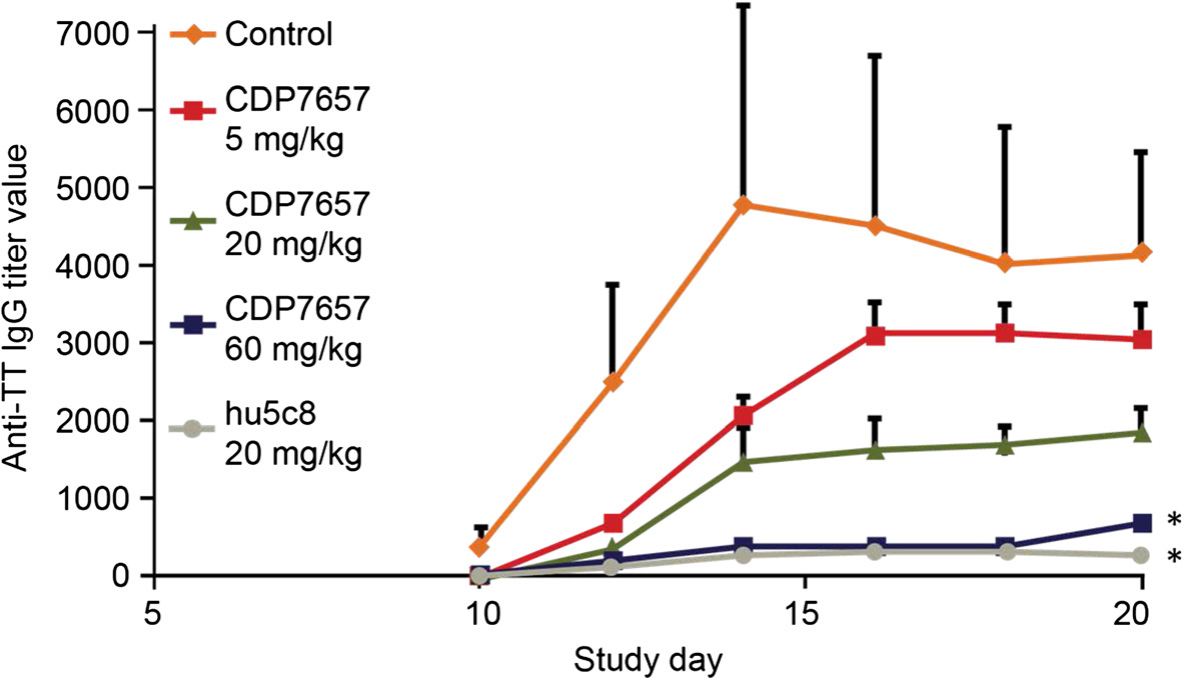
Inhibition of humoral immune response in Cynomolgus monkeys: CDP7657 dose response. CDP7657 (various doses) was compared with a single dose of hu5c8 (20 mg/kg). Animals were administered a single dose of antibody or saline i.v. on day 1 and challenged with tetanus toxoid (*TT*) 4 h later. Data are expressed as the mean anti-TT IgG titer ± standard deviation; *P <0.05 (one-way analysis of variance), compared with control, was considered to be statistically significant. Maximum concentration (C_max_) and area under the concentration–time curve from time 0 until 19 days (AUC_0–19_) values were calculated for each tested dose of antibody, day 19 being the last day anti-TT response was measured after anti-CD40 ligand administration. C_max_ was 163, 704 and 2,088 μg/mL for 5, 20 and 60 mg/kg doses of CDP7657, respectively. AUC_0–19_ was 713, 2476 and 10,488 μg/day/mL for 5, 20 and 60 mg/kg CDP7657, respectively. C_max_ and AUC_0–19_ for hu5c8 (20 mg/kg) were 414 and 3,186 μg/day/mL, respectively

A single-dose study was conducted to assess the efficacy of CDP7657 at three dose levels (5, 20 and 60 mg/kg i.v.), compared with hu5c8 at 20 mg/kg i.v., a dose shown to be effective in previous studies with this antibody [23]. The pharmacokinetics were considered to be typical for the antibody constructs used, and were dose-proportional. Similar overall exposures were achieved with CDP7657 and hu5c8 at the same dose of 20 mg/kg i.v (Fig. 1 legend).

**Fig. 2:**
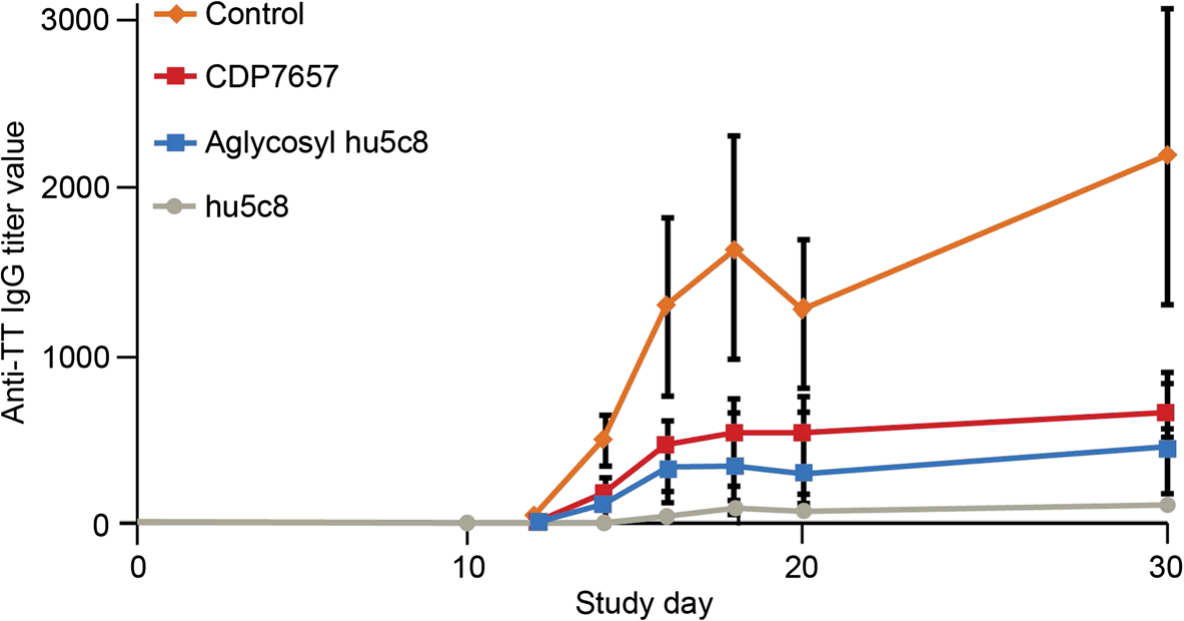
Inhibition of humoral immune response in Cynomolgus monkeys: comparison of antibody formats. Animals received a single i.v. dose of antibody or saline 2 days prior to administration of tetanus toxoid (*TT*); the doses were 20 mg/kg for the IgG antibodies and 40 mg/kg for the PEGylated formats (n = 6 for all groups). The IgG anti-TT titers in plasma samples were assessed from days 10 to 31

The serum IgG responses elicited by TT provocation were inhibited dose-dependently by CDP7657, and the inhibition was statistically significant with 60 mg/kg i.v. (*P* <0.05) (Fig. 1). The degree of inhibition of the IgG response seen with CDP7657 at 60 mg/kg i.v. was similar to that achieved with hu5c8 at 20 mg/kg i.v. A similar pattern of inhibition was observed when the secondary immune response was assessed after administration of a second dose of antibody to the animals and re-challenge with TT (see Additional file 1). A reduction in potency was seen with an aglycosyl derivative of hu5c8 compared to hu5c8 (Fig. 2). In this molecule, a single amino acid change in the Fc region prevents normal N-glycosylation of the antibody [23]. This change does not alter the affinity of the antibody for CD40L, but does ablate antibody effector function including FcγR binding. Aglycosyl hu5c8 was shown to have a similar potency to CDP7657 in this model. Taken together, these data demonstrate that Fc function plays a role in the potency of anti-CD40L antibodies in this *in vivo* model, although equivalent efficacy was achieved with CDP7657 when dosed at 60 mg/kg.

### A murine anti-CD40L monovalent Fab' PEG antibody fragment inhibits disease in SLE-prone NZB/W F1 mice

**Fig. 3:**
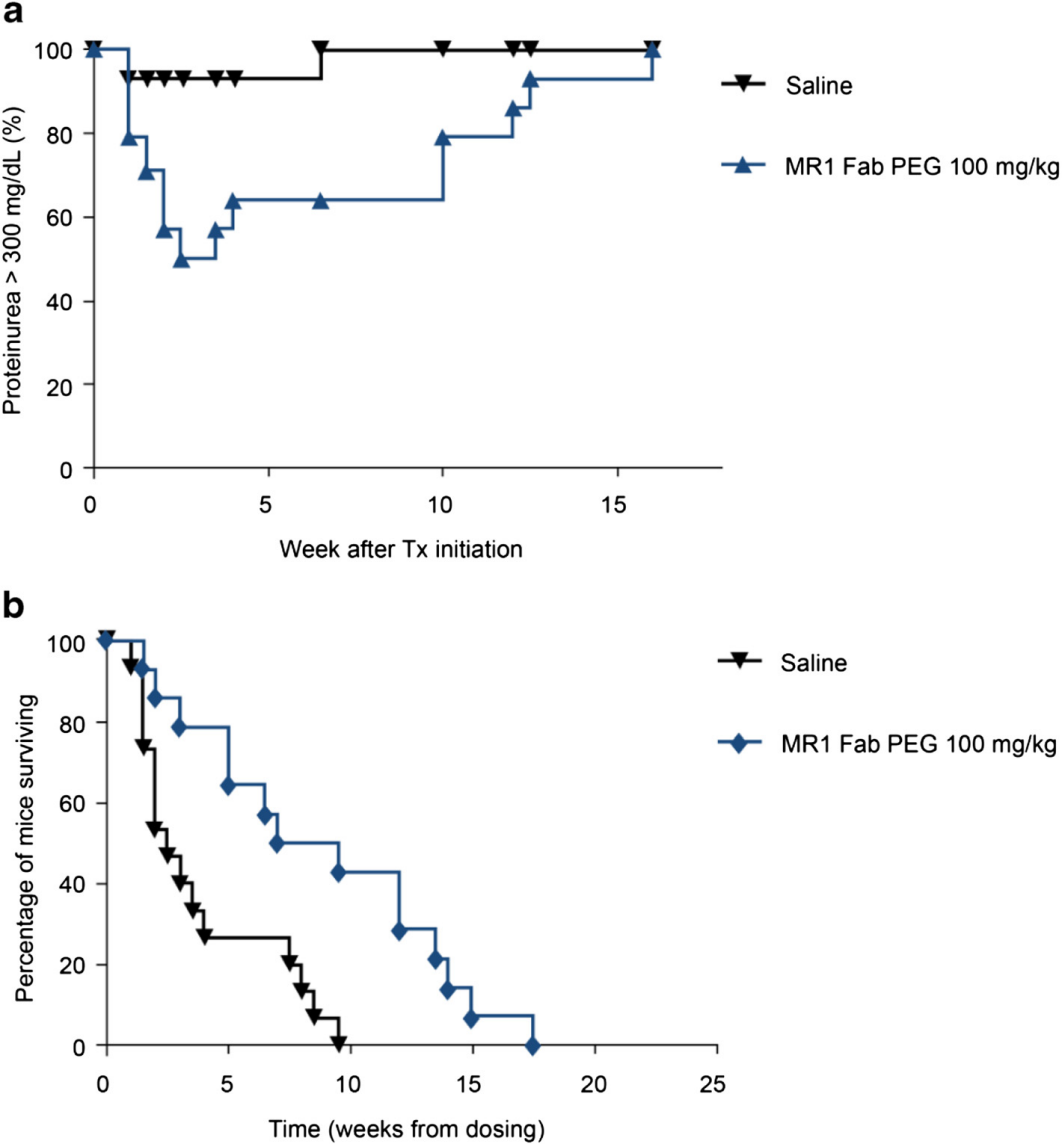
Disease remission and increased survival after therapeutic dosing with MR1 Fab' polyethylene glycol (*PEG*) in NZB/NZW mice. **a** Remission and relapse of proteinuria levels in NZB/W mice after a single dose of 100 mg/kg MR1 Fab' PEG (n = 14) compared to saline control (n = 15). Data are expressed as the percentage of animals scoring ≥300 mg/dL proteinuria, with remission classified as two consecutive scores ≤100mg/dL proteinuria; *P* = 0.0146 (Mann–Whitney *U* test) compared with saline control. **b** Percentage of mice surviving in each group over time; *P* <0.0031 (log-rank test) compared to saline control, and was considered to be statistically significant. *Tx* therapy

To assess whether an anti-CD40L antibody construct lacking an Fc region retains efficacy in experimental models of autoimmune disease, a PEGylated monovalent Fab' anti-mouse antibody, MR1 Fab' PEG, was generated. Female NZB/NZW F1 mice spontaneously develop an autoimmune disease, which has many of the pathological features of human SLE. MR1 Fab' PEG demonstrates profound inhibition in this model when dosed prophylactically (i.e., before disease onset), but has also been assessed under more stringent conditions using a therapeutic dosing regimen whereby mice were administered antibodies only after demonstrating high levels of proteinuria (>300 mg/dL). Under these conditions, treatment with a single dose of MR1 Fab' PEG resulted in protection from disease, with 50 % of animals entering remission (proteinuria level ≤100 mg/dL measured on two consecutive occasions) compared to a saline control group, resulting in increased survival of the treated animals (Fig. 3). These data show that a monovalent PEGylated anti-CD40L antibody fragment profoundly affects disease remission in an experimental model of SLE, as had previously been demonstrated for full-length anti-CD40L antibodies [24].

### Platelet activation by anti-CD40L antibodies requires FcγRIIa interaction

As well as confirming that CDP7657 was efficacious in both rodent and Cynomolgus monkey models, it was important to determine whether this construct mitigated the Fc-mediated platelet activation believed to be responsible for the prothrombotic events observed with hu5c8. Using platelet function tests, the possible prothrombotic activity of hu5c8, aglycosyl hu5c8 and CDP7657 was explored using human and Rhesus monkey blood.

**Fig. 4:**
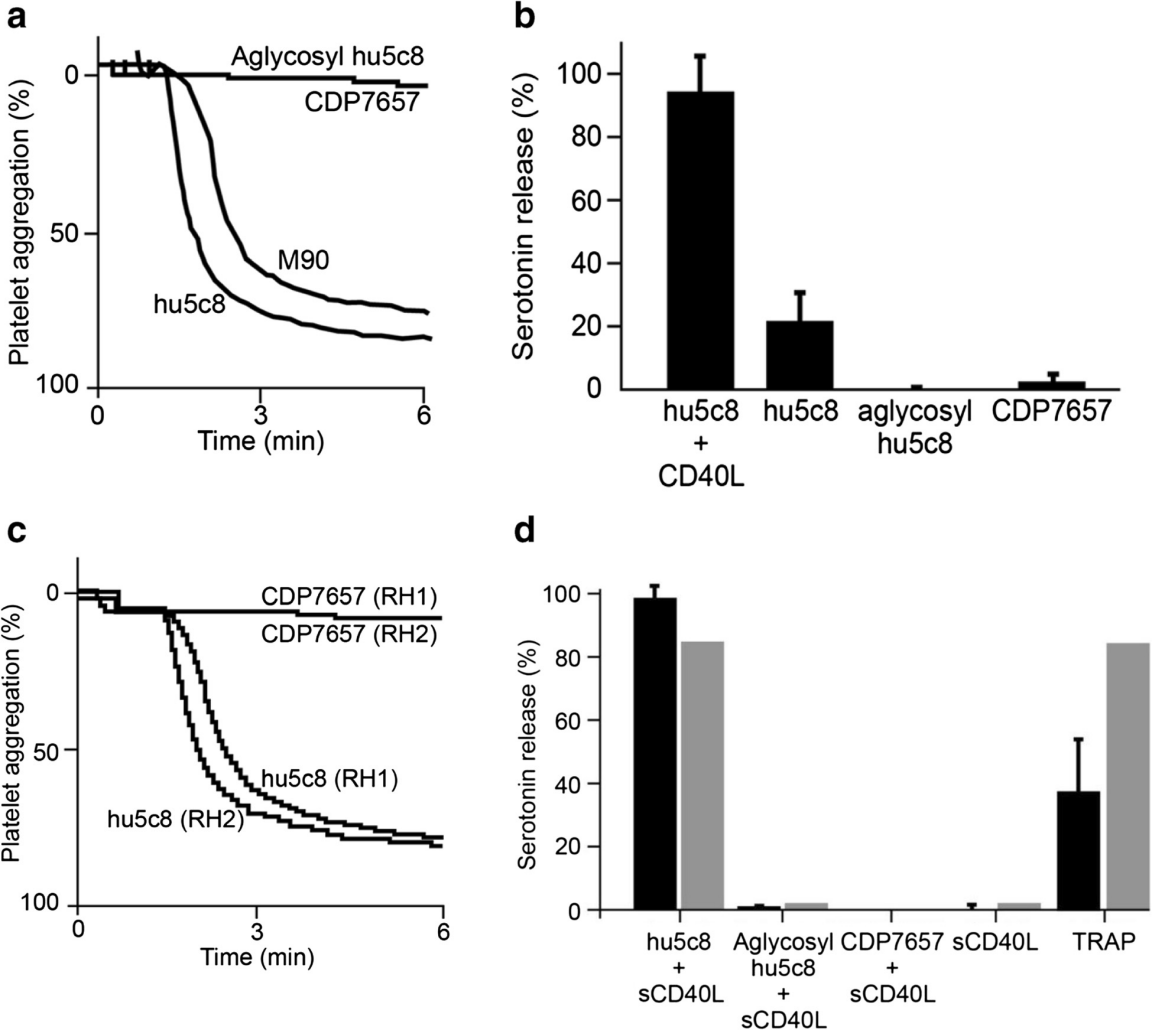
Platelet activation by anti-CD40 ligand (anti-*CD40L*)antibodies requires FcγRIIa interaction. **a** *In vitro* platelet aggregation isolated from humans. Preformed immune complex (IC) (anti-CD40L antibodies plus soluble CD40 ligand (*sCD40L*)) was incubated with isolated platelets from four human volunteers; representative data from one volunteer are shown. Traces show the percentage of platelet aggregation over a 6-minute time period from one donor, but is representative of four donors. **b** *In vitro* dense granule release from human platelets. Anti-CD40L antibodies (final concentration 500 nM, with/without sCD40L) were incubated with isolated human platelets (n = 4), and ^14^C-radiolabeled serotonin release was measured. Data are plotted as mean percentage serotonin release (average of the means of four donors (SRA)). Error bars are standard deviation for the mean percentage SRA values of the four donors. **c** *In vitro* platelet aggregation isolated from Rhesus monkeys. Preformed IC was incubated with isolated platelets from two Rhesus monkeys (*RH1* and *RH2*). Traces show the percentage of platelet aggregation over a 6-minute time period from one donor, but is representative of two donors. **d** *In vitro* dense granule release from Rhesus monkey platelets. Preformed IC or antibody solutions (final concentrations 500 nM) were incubated with isolated platelets from four Rhesus monkeys (*black bars*) or one healthy human donor (*gray bars*), and ^14^C-radiolabeled serotonin release was measured. Thrombin receptor agonist peptide (*TRAP*) (100 μM) was included as a positive control. Data are plotted as in **b**.

In washed platelets from human healthy donors, immune complex (IC) consisting of hu5c8 with sCD40L (hu5c8 IC) rapidly caused complete aggregation, whereas aglycosyl hu5c8 IC and CDP7657 IC were completely inactive (Fig. 4a). Hu5c8, aglycosyl hu5c8 and CDP7657 were all inactive in the absence of sCD40L, as was sCD40L alone (see Additional file 2). M90, another anti-human CD40L antibody, also induced platelet aggregation in an intact murine IgG format, when combined with sCD40L. Additionally, hu5c8 IC consistently induced a strong serotonin dense granule release from isolated platelets (Fig. 4b) but CDP7657 and aglycosyl hu5c8 were completely inactive.

Comparable experiments performed using Rhesus monkey blood samples were consistent with the human findings. As shown in Fig. 4c, hu5c8 IC caused complete aggregation of washed Rhesus monkey platelets, whereas CDP7657 IC had no aggregatory effect. Similarly, hu5c8 IC induced a strong serotonin dense granule release from Rhesus monkey platelets (Fig. 4d). Aglycosyl hu5c8 IC and CDP7657 IC, in addition to hu5c8, aglycosyl hu5c8, CDP7657 or sCD40L alone were completely inactive.

Overall, these data suggest that aglycosyl hu5c8 IC and CDP7657 IC do not induce platelet aggregation in both human and Rhesus monkey test systems; the same was true even when Rhesus monkey platelets were primed with sub-optimal ADP (See Additional file 3). These data are consistent with earlier reports showing that CD40L antibodies require Fc-mediated signaling through FcγRIIa to activate platelets [12] and that aglycosyl hu5c8 (incapable of binding FcR) and antibody fragments lacking an Fc region are without activity in these assay systems.

### Lack of evidence of TEs in Rhesus monkeys with CDP7657

**Fig. 5:**
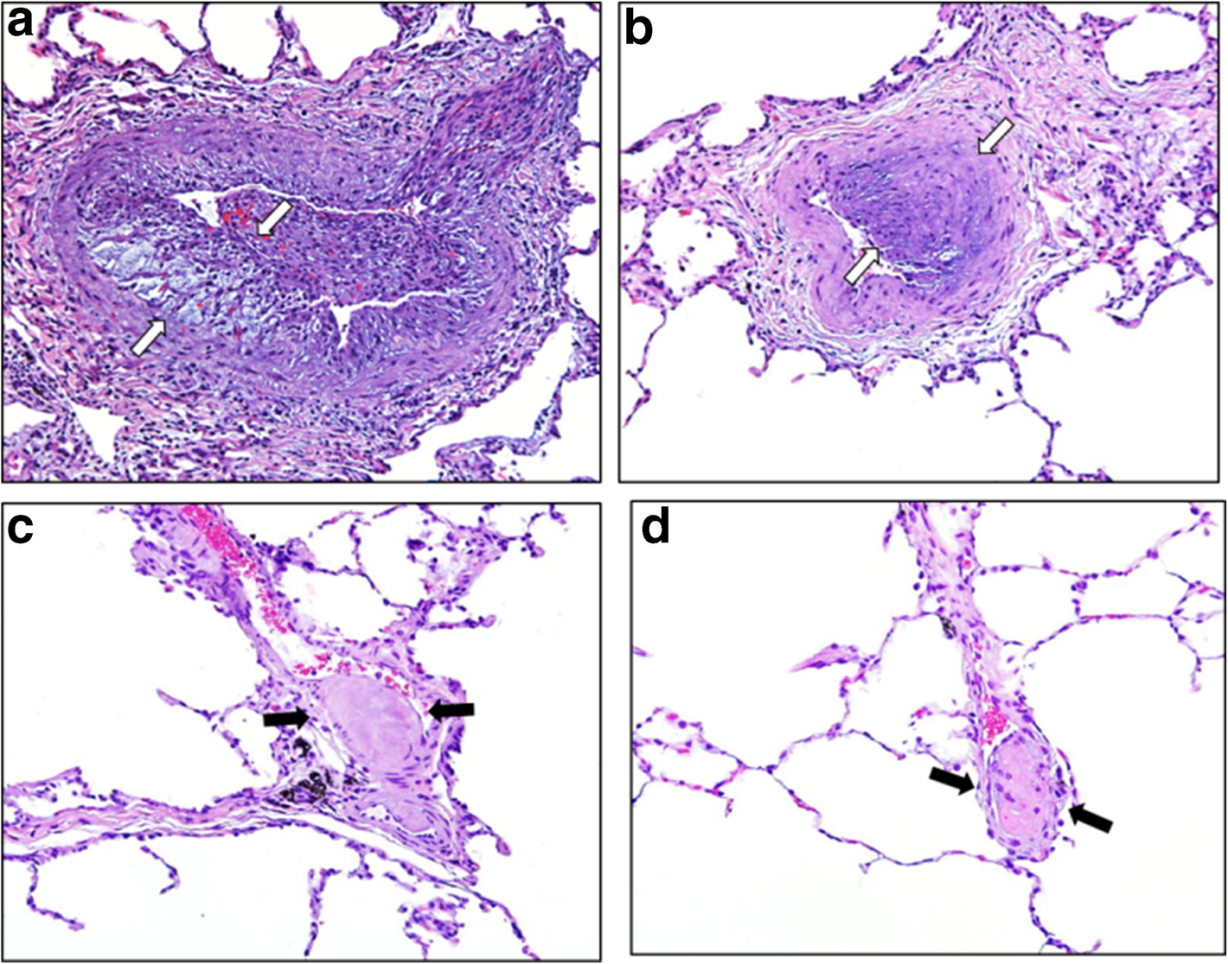
Histopathology of Rhesus monkey lungs showed no findings attributable to CDP7657. Representative hematoxylin and eosin stained sections of pulmonary arteries/arterioles from Rhesus monkeys that received hu5c8 (**a-b**), vehicle control (**c**) or CDP7657 (**d**). In **a** and **b** arterial thrombus formation is accompanied by marked, irregular thickening of the intimal layer (*arrows*). Intimal thickening is mediated by in-migration of smooth muscle, deposition of pale basophilic fibrillar extracellular matrix and cleft formation. Vessel lumina contain fibrinocellular debris and entrapped red blood cells. In **c** and **d** fibrin microthrombi adhere to the endothelial lining of thin-walled arterioles (*arrows*). Under normal conditions, microthrombi are formed and lysed continually within the venous circulatory system. Small pulmonary arterial vessels and capillaries trap thrombi and prevent them from obstructing the vital coronary, cerebral and renal arteries. These observations were isolated, infrequent findings

Given that CDP7657 did not lead to activation of platelets from either humans or Rhesus monkeys, an evaluation to determine whether this would translate to a lack of TEs *in vivo* was undertaken. A head-to-head, 8-week i.v. study to compare the thromboembolic potential of CDP7657 and hu5c8 was conducted in Rhesus monkeys. Hu5c8 at 50 mg/kg/week i.v. produced widespread pulmonary arterial intravascular thrombosis and/or intimal hyperplasia and associated secondary pathology changes in the lungs (Table 3; Fig. 5a and b). By contrast, CDP7657 and other anti-CD40L antibody constructs lacking a functional Fc region, also administered at 50 mg/kg/week i.v., were associated with minimal pulmonary changes that were comparable in incidence and severity to those found in saline-treated control animals (Table 3; Fig. 5c and d). In two out of eight hu5c8-treated animals, there was a treatment-related decrease in platelet count (56 % and 42 % on day 37, compared with pre-treatment levels) but this was not observed in any CDP7657-treated animal.

**Table 3:** Thromboembolic potential of different anti-CD40L antibody formats in a comparative 8-week study in Rhesus monkeys

Dose group (n)	Total animals affected (%)	Total affected lung sections	Total examined lung sections	Lung sections affected (%)
Historic saline controls	7 (50)	10	406	2.5
(n = 14)^a^				
Concurrent saline control	1 (25)	1	116	0.9
(n = 2/sex)^a^				
CDP7657	3 (37.5)	4	232 (203)^c^	1.7 (2.0)^c^
(n = 4/sex)^b^				
Aglycosyl hu5c8	3 (37.5)	3	232	1.3
(n = 4/sex)				
Hu5c8	5 (62.5)	41	232	17.6
(n = 4/sex)				

Thrombembolic events were defined as the occurrence of thrombus, an organizing thrombus or intimal hyperplasia. ^a^Historic control group of Rhesus monkeys that had received i.v. saline for up to 6 months; ^b^one animal died on day 2, which was unrelated to CDP7657 treatment; ^c^values in parentheses do not include animals removed from the study for humane reasons unrelated to the study

Based on the encouraging *in vitro* and *in vivo* data generated with CDP7657, a longer study with higher dose levels of CDP7657 was undertaken. In this study, Rhesus monkeys were dosed weekly for 3 months with CDP7657 at doses up to 200 mg/kg i.v. No gross or histopathologic findings attributable to CDP7657 were observed in the lungs of treated animals, either at the end of the 3-month treatment period or during a period of up to 6 months following the end of dosing (Table 4). Microscopic thrombi (including mature organizing thrombi) with a similar very low incidence and distribution were observed in both CDP7657-treated and saline-treated control animals. These were considered to be incidental, unrelated to CDP7657, and consistent with background levels observed in historical experiments performed with Rhesus monkeys. Furthermore, no qualitative morphological differences between thrombi were seen between CDP7657-treated and saline-treated control groups. At the end of the no-treatment recovery period, microscopic pulmonary thrombi were found in the saline-treated control and CDP7657-treated groups, with a similar low incidence to those found at the end of the dosing period, indicating no association with CDP7567 treatment. Thus, even after prolonged high-dose exposure, there is no evidence that CDP7657 is able to induce TEs *in vivo.* In addition, no evidence of other toxicity was demonstrated in this study.

**Table 4:** Thromboembolic potential of CDP7657 in an extended 3-month study in Rhesus monkeys

Dose group	Total animals affected (%)	Total affected lung sections	Total examined lung sections^a^	Lung sections affected (%)
Control^b^	10 (50)	11	581	1.9
(n = 10/sex)				
20 mg/kg	6 (60)	9	290	3.1
(n = 5/sex)				
50 mg/kg	4 (40)	4	294	1.4
(n = 5/sex)				
200 mg/kg	3 (33)	3	261	1.2
(n = 5/sex)				

Thromboembolic events defined as the occurrence of a thrombus or an organizing thrombus (intimal hyperplasia was not observed in this study); ^a^total number of lung sections examined in each group = 29/animal; ^b^treated with i.v. saline

The prothrombotic antibody hu5c8 has a fully functional Fc region and is considered to be capable of antigen cross-linking, whereas CDP7657, an agent that is not associated with thrombus formation, does not have an Fc region and is only capable of monovalent binding. Further investigation of the mechanism by which hu5c8 promotes thrombus formation showed that treatment with the aglycosyl form of hu5c8 was not associated with thrombus formation (Table 3).

## Discussion

The key role played by the CD40–CD40L axis in regulating T-cell/B-cell interactions makes it a particularly attractive target for the treatment of inflammatory and autoimmune diseases. However, the promise of CD40L-specific antibodies has been challenged by the association of hu5c8 with TEs [15, 18]. More recent work has suggested that these unexpected side effects are likely dependent upon the anti-CD40L antibody carrying a functional Fc region [12]. As shown in the pre-clinical studies presented here, CDP7657, a novel high-affinity anti-CD40L PEGylated monovalent Fab' antibody fragment that lacks an Fc region, dose-dependently inhibits the immune response to TT, but does not induce TEs in monkeys.

In this study, CDP7657 and aglycosyl hu5c8, antibody constructs that lack a functional Fc region, strongly inhibited the humoral response to TT, although they had reduced activity compared with hu5c8 (even allowing for plasma exposure), which almost certainly reflects some role for an active Fc in this particular model. Interestingly, aglycosyl hu5c8 has been shown to be effective in the TT immune response model as well as in SLE and experimental autoimmune encephalomyelitis mouse models, but was ineffective in both renal and allograft transplantation models [23, 25]. This suggests that the contribution of Fc-effector function of anti-CD40L antibodies may vary depending on the immune challenge [23]. However, in the current study 60 mg/kg CDP7657 was still able to abrogate the immune response to the TT antigen to the same degree as hu5c8, despite not possessing an Fc.

As has been highlighted previously, anti-CD40L antibodies are particularly effective in mouse SLE models, showing profound effects on survival as well as proteinuria, autoantibodies and immune cell populations [7]. Interestingly, a more recent study examined disease and mechanistic endpoints in a CD40L^−/−^ NZB SLE mouse model [26]. CD40L^−/−^ led to abrogated production of autoantibodies, decreases in activated T cells and memory effector cells, lack of germinal center development and reduced plasmablast number. These data corroborate data generated with anti-CD40L antibodies, illustrating that CD40L has an important role in promoting pathogenic autoantibody production and kidney disease in NZB mice, and highlight the potential therapeutic benefit of CD40L blockade as a treatment for autoimmune diseases, particularly those driven by autoantibodies.

In this study, disease remission was seen with a murine anti-CD40L PEGylated monovalent Fab' antibody (MR1 Fab' PEG) in the NZB/W mouse SLE model, using a therapeutic dosing regimen where mice received antibodies only after they had displayed severe proteinuria. Most work that has been published using this model utilized a prophylactic dosing regimen, where treatment was started prior to disease onset. Therapeutic efficacy has been demonstrated previously with the full-length MR1 anti-CD40L antibody [24], but the data presented here suggest that CDP7657, lacking any Fc-function, may have the potential to be an effective therapy in lupus patients with demonstrable disease.

Given the previous history of anti-CD40L antibody evaluation in the clinic, a key requirement for progressing new entities targeting CD40L has been to understand the mechanisms for induction of TE and to design a suitable *in vivo* model that could demonstrate that the risk of TEs associated with anti-CD40L antibodies has been eliminated. In our studies of isolated human and Rhesus monkey platelets *in vitro*, activation occurred with hu5c8 IC but not with complexes formed with CDP7657 or other antibody formats lacking an Fc region. Furthermore, although hu5c8 was shown to activate human platelets *in vitro* when it possessed an active Fc moiety, it was without activity in its aglycosyl form in these assay systems. Likewise, CDP7657 had no platelet-activating activity in human or Rhesus monkey platelet assays.

These observations are consistent with other studies suggesting a critical role of the IgG Fc regions of anti-CD40L antibodies in mediating platelet aggregation [20]. *In vitro* studies showed that hu5c8/CD40L IC, but not aglycosyl hu5c8 /CD40L IC, activated platelets and that an anti-FcγRIIa monoclonal antibody can block the activation of platelets triggered by intact anti-CD40L/CD40L IC [12, 20]. As FcγRIIa is the only IgG Fc receptor detectably expressed on the surface of platelets [27], the involvement of other IgG Fc receptors in these observations is unlikely. More recently, the role of FcγRIIa in IC-triggered thrombosis was confirmed in transgenic mice [21] where anti-human CD40L/sCD40L IC triggered a thrombotic response in mice transgenic for human FcγRIIa, but not in wild-type mice. Furthermore, the thrombotic effect observed in FcγRIIa transgenic mice could be triggered by IC comprising soluble mouse CD40L and the anti-mouse CD40L antibody MR1 carrying a human Fc region (hMR1), but not by IC of soluble mouse CD40L and aglycosyl hMR1. Overall, the data suggest a mechanism whereby co-engagement of CD40L and FcR by an intact antibody triggers activation of platelets, possibly following cross-linking events and triggering of signaling; in theory, this could happen in *cis*, with co-engagement of CD40L and FcR on the same platelet surface, or in *trans*, mediated by platelet–platelet interactions. *In vitro* studies published recently with a domain Ab anti-CD40L antibody, which lacks a functional Fc, similarly concluded that the Fc was essential for platelet activation [28]. These authors also showed that an anti-mouse CD40L domain Ab was efficacious in a number of mouse models of autoimmune disease.

We have previously published work that suggests that the Rhesus monkey is a relevant and sensitive pre-clinical model for induction of TEs by anti-CD40L IgG_1_ antibodies in humans [19]. In the current study, hu5c8 treatment produced extensive pulmonary TEs and pulmonary vasculopathy in Rhesus monkeys. In contrast, no treatment-related adverse findings were observed with CDP7657 or aglycosyl hu5c8 and the pulmonary findings observed in CDP7657-treated animals were similar to those observed in saline-treated control animals. No TEs attributable to CDP7657 (using doses up to 200 mg/kg i.v.) were observed in a 3-month repeat dose safety study, either during the treatment period or after a 6-month treatment-free period, again in contrast to hu5c8. This is the first demonstration that a CD40L antibody lacking the Fc domain, and thus no longer able to activate platelets *in vitro*, has mitigated the risk of inducing TEs *in vivo* in Rhesus monkeys.

In all dose groups, including saline control animals, 33 − 60 % of animals had small, occasional, solitary pulmonary micro-thrombi that were considered to be incidental to injection. It is suspected that these incidental thrombi may originate from the injection site and/or blood sampling venipuncture sites, arising secondary to minor venous trauma [29] and were only observed because of extensive lung tissue sectioning and high examination scrutiny.

Published data suggest that the blockade of CD40L *per se* would not be expected to cause TE events. Studies in CD40L^−/−^ knockout mice showed protection against thrombosis by genetic deficiency in the CD40L pathway in which accelerated thrombus formation was completely abrogated in arterioles and partially abrogated in venules in CD40L^−/−^ mice [30]. Exogenous sCD40L reversed this protection. In another study, platelets from CD40L-deficient mice demonstrated reduced shear-induced platelet aggregation [31]. Elevated sCD40L has also been linked to an increased risk of atherothrombosis [32]. It should be stressed that mouse platelets, in contrast to human and non-human primate platelets, do not express FcRs and, therefore, these published studies suggest that blocking CD40L alone would not contribute to an increased incidence of thrombosis and possibly would even suppress thrombosis under certain conditions. Indeed, in our studies, the pulmonary thrombovasculopathy observed with hu5c8 in Rhesus monkeys was dependent on the presence of a functional Fc region, and was not induced by CD40L blockade alone.

## Conclusions

The present studies have demonstrated that anti-CD40L antibodies require a functional Fc region in order to induce TEs in Rhesus monkeys*.* CDP7657, a novel high-affinity anti-CD40L PEGylated monovalent Fab' antibody without an Fc region, effectively inhibits CD40L-dependent immune responses. This, in conjunction with the lack of TE and Fc-mediated platelet activation, supports the clinical evaluation of this antibody in humans for the treatment of SLE and other autoimmune diseases.

## Additional files

Additional file 1:
**Inhibition of secondary immune response in Cynomolgus monkeys.** CDP7657 at 5 or 20 mg/kg (60 mg/kg was not evaluated in this study) was compared with hu5c8 at 20 mg/kg. Animals were administered a single dose of antibody or i.v saline. and challenged with tetanus toxoid (*T*T) on day 1; they then received a second dose of antibody and TT on day 30. Data are expressed as the mean anti-TT IgG titer ± standard deviation; approximately 50 % inhibition was observed at 20 mg/kg CDP7657, although this was not statistically significant. ****P* <0.001 (one-way analysis of variance) compared with control; *NS* not significant. (PDF 66 kb) Additional file 2:
**Hu5c8, aglycosyl hu5c8 and CDP7657 are all inactive in the absence of sCD40L, as was sCD40L alone.** *In vitro* platelet aggregation assay of human washed platelets. **A** Hu5c8 (*blue*), CDP7657 (*black*) and aglycosyl hu5c8 (*red*) did not cause platelet aggregation when introduced to platelets without recombinant human soluble CD40L (rhCD40L). **B** rhCD40L alone (10 μg/ml) was also inactive. (PDF 180 kb) Additional file 3:
**CDP7657 immune complex (** ***IC*** **) and aglycosyl hu5c8 IC do not induce platelet aggregation in rhesus monkey platelets both in the absence of presence of sub-aggregatory amounts of the platelet agonist (ADP).** *In vitro* aggregation assay of rhesus monkey washed platelets. **A** CDP7657 IC did not aggregate rhesus monkey washed platelets in the absence (*blue* and *red*), or presence (*black* and *green*) of ADP. **B** Similarly, aglycosyl hu5c8 IC did not aggregate rhesus monkey washed platelets in the absence (*blue* and *red*), or presence (*black* and *green*) of ADP. (PDF 171 kb)

## Abbreviations

ADP: adenosine diphosphate
ANOVA: analysis of variance
AUC_0–19_: area under the concentration–time curve from time 0 until 19 days
CD40L: CD40 ligand
C_max_: maximum concentration
ELISA: enzyme-linked immunosorbent assay
FcR: Fc receptor
GLP: Good Laboratory Practice
HRP: horseradish peroxidase
IC: immune complex
ICAM-1: intercellular adhesion molecule-1
i.m.: intramuscular
i.v.: intravenous
K_D_: equilibrium dissociation constant
kDa: kiloDaltons
NZB/W: New Zealand black/white
PE: phycoerythrin
PEG: polyethylene glycol
rhCD40L: recombinant human soluble CD40L
s.c: subcutaneous
sCD40L: soluble CD40L
SLE: systemic lupus erythematosus
SRA: serotonin release assay
TE: thrombotic events
TRAP: thrombin receptor agonist peptide
TT: tetanus toxoid
Tx: treatment
