# Supplementary material for: CDP7657, an anti-CD40L antibody lacking an Fc domain, inhibits CD40L-dependent immune responses without thrombotic complications: an in vivo study
Source: Arthritis Res Ther. 2015 Sep 3;17(1):234. doi: 10.1186/s13075-015-0757-4 (PMC4558773; doi:10.1186/s13075-015-0757-4)
Supplement: Additional file 1: — Inhibition of secondary immune response in Cynomolgus monkeys. CDP7657 at 5 or 20 mg/kg (60 mg/kg was not evaluated in this study) was compared with hu5c8 at 20 mg/kg. Animals were administered a single dose of antibody or i.v saline. and challenged with tetanus toxoid (TT) on day 1; they then received a second dose of antibody and TT on day 30. Data are expressed as the mean anti-TT IgG titer ± standard deviation; approximately 50 % inhibition was observed at 20 mg/kg CDP7657, although this was not statistically significant. ***P <0.001 (one-way analysis of variance) compared with control; NS not significant. (PDF 66 kb) [file 13075_2015_757_MOESM1_ESM.pdf]

Inhibition of secondary immune response in Cynomolgus monkeys

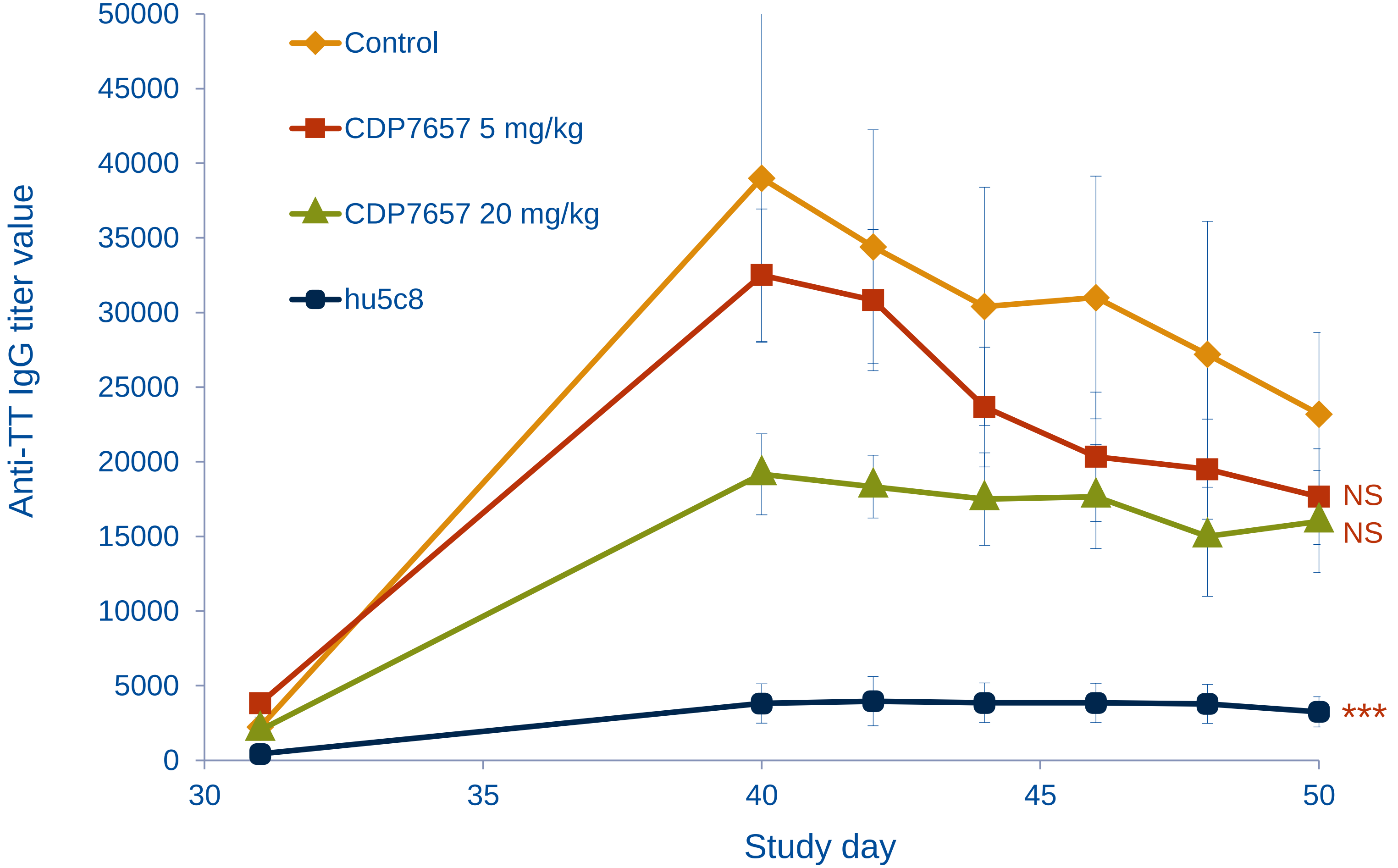

CDP7657 at 5 or 20 mg/kg (60 mg/kg was not evaluated in this study) was compared with hu5c8 at 20 mg/kg. Animals were administered a single dose of antibody or saline i.v. and challenged with TT on day 1; they then received a second dose of antibody and TT on day 30. Data are expressed as the mean anti-TT IgG titer ± standard deviation; Approximately 50% inhibition was observed at 20 mg/kg CDP7657, although this did not reach statistical significance. ***P < 0.001 (one-way ANOVA), compared with control.
