# Supplementary material for: CDP7657, an anti-CD40L antibody lacking an Fc domain, inhibits CD40L-dependent immune responses without thrombotic complications: an in vivo study
Source: Arthritis Res Ther. 2015 Sep 3;17(1):234. doi: 10.1186/s13075-015-0757-4 (PMC4558773; doi:10.1186/s13075-015-0757-4)
Supplement: Additional file 2: — Hu5c8, aglycosyl hu5c8 and CDP7657 are all inactive in the absence of sCD40L, as was sCD40L alone. In vitro platelet aggregation assay of human washed platelets. A Hu5c8 (blue), CDP7657 (black) and aglycosyl hu5c8 (red) did not cause platelet aggregation when introduced to platelets without recombinant human soluble CD40L (rhCD40L). B rhCD40L alone (10 μg/ml) was also inactive. (PDF 180 kb) [file 13075_2015_757_MOESM2_ESM.pdf]

Hu5c8, aglycosyl hu5c8 and CDP7657 are all inactive in the absence of sCD40L, as was sCD40L alone

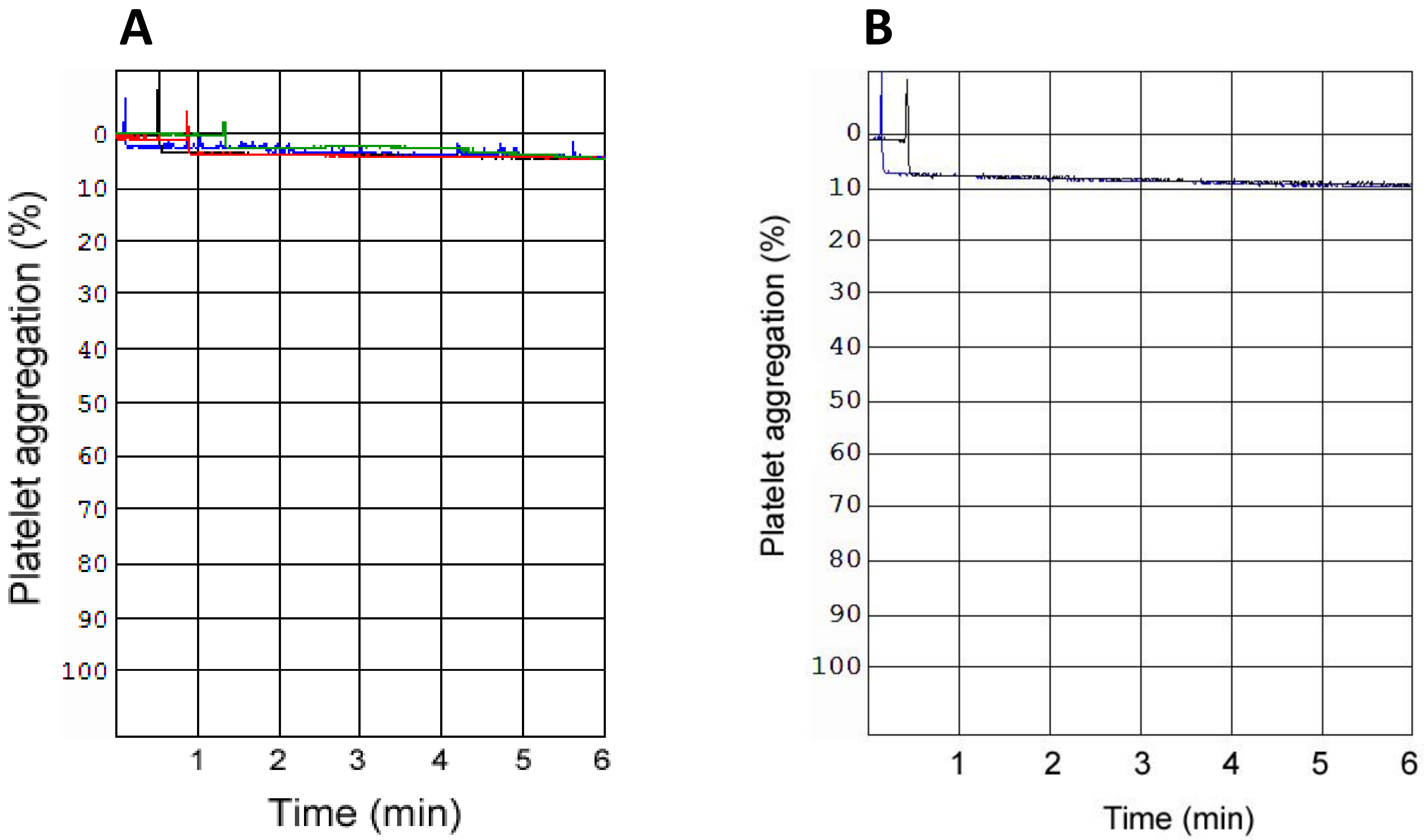

In vitro platelet aggregation assay of human washed platelets. **(A)** Hu5c8 (blue), CDP7657 (black) and aglycosyl hu5c8 (red) did not cause platelet aggregation when introduced to platelets without recombinant human soluble CD40L (rhCD40L). **(B)** rhCD40L alone (10 µg/ml) was also inactive.
