# Supplementary material for: CDP7657, an anti-CD40L antibody lacking an Fc domain, inhibits CD40L-dependent immune responses without thrombotic complications: an in vivo study
Source: Arthritis Res Ther. 2015 Sep 3;17(1):234. doi: 10.1186/s13075-015-0757-4 (PMC4558773; doi:10.1186/s13075-015-0757-4)
Supplement: Additional file 3: — CDP7657 immune complex ( IC ) and aglycosyl hu5c8 IC do not induce platelet aggregation in rhesus monkey platelets both in the absence of presence of sub-aggregatory amounts of the platelet agonist (ADP). In vitro aggregation assay of rhesus monkey washed platelets. A CDP7657 IC did not aggregate rhesus monkey washed platelets in the absence (blue and red), or presence (black and green) of ADP. B Similarly, aglycosyl hu5c8 IC did not aggregate rhesus monkey washed platelets in the absence (blue and red), or presence (black and green) of ADP. (PDF 171 kb) [file 13075_2015_757_MOESM3_ESM.pdf]

CDP7657 IC and aglycosyl hu5c8 IC do not induce platelet aggregation in rhesus monkey platelets both in the absence of presence of sub-aggregatory amounts of the platelet agonist (ADP)

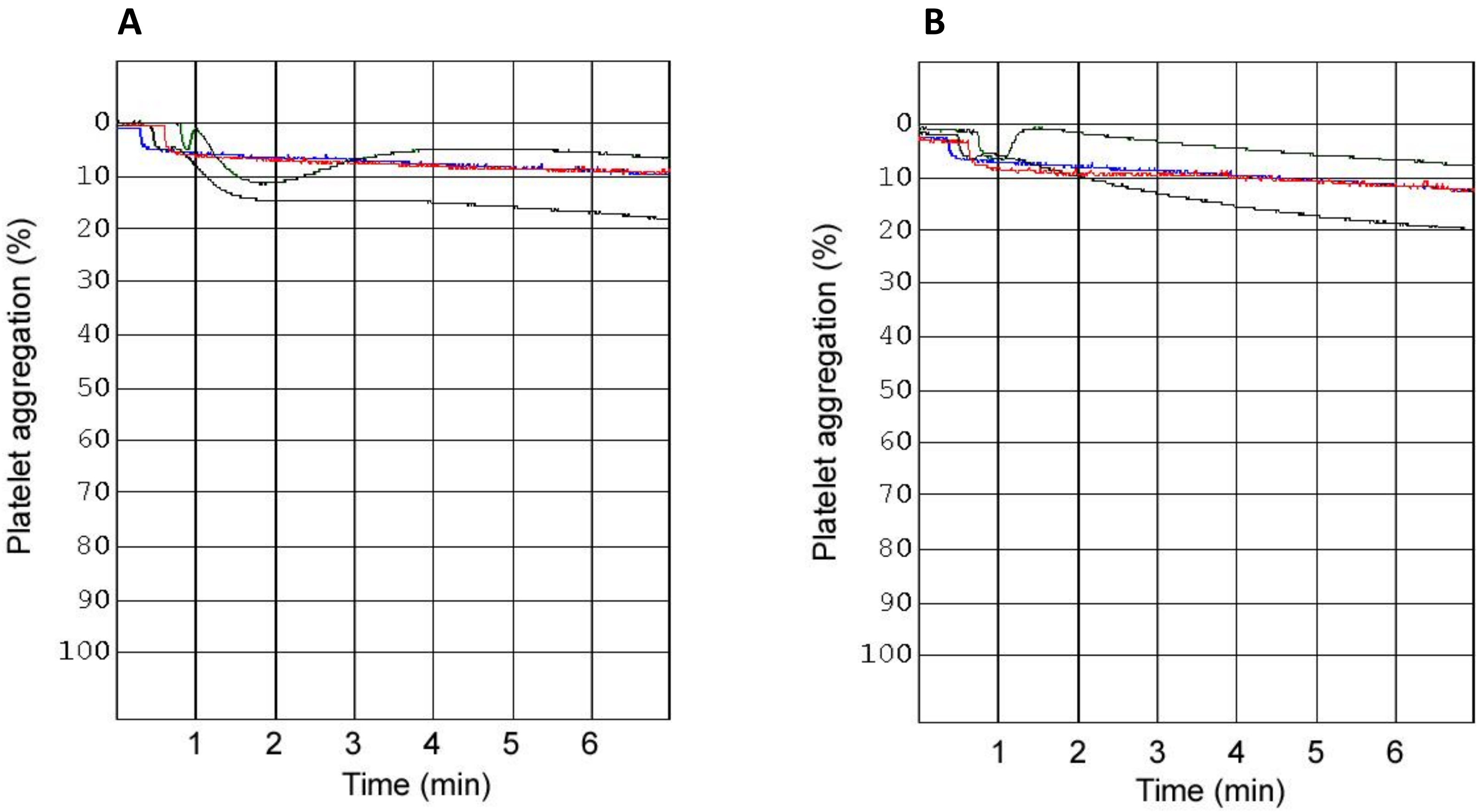

In vitro aggregation assay of rhesus monkey washed platelets. **(A)** CDP7657 IC did not aggregate rhesus monkey washed platelets in the absence (blue and red), or presence (black and green) of ADP. **(B)** Similarly, aglycosyl hu5c8 IC did not aggregate rhesus monkey washed platelets in the absence (blue and red), or presence (black and green) of ADP.
